# *Ghd2,* a *CONSTANS*-like gene, confers drought sensitivity through regulation of senescence in rice

**DOI:** 10.1093/jxb/erw344

**Published:** 2016-09-16

**Authors:** Juhong Liu, Jianqiang Shen, Yan Xu, Xianghua Li, Jinghua Xiao, Lizhong Xiong

**Affiliations:** National Key Laboratory of Crop Genetic Improvement and National Center of Plant Gene Research (Wuhan), Huazhong Agricultural University, Wuhan 430070, China

**Keywords:** ARID, *CO*-like, drought, Ghd2, *Oryza sativa*, senescence

## Abstract

*CONSTANS* (*CO*)-like genes have been intensively investigated for their roles in the regulation of photoperiodic flowering, but very limited information has been reported on their functions in other biological processes. Here, we found that a *CO*-like gene, *Ghd2* (*Grain number, plant height, and heading date2*), which can increase the yield potential under normal growth condition just like its homologue *Ghd7*, is involved in the regulation of leaf senescence and drought resistance. *Ghd2* is expressed mainly in the rice (*Oryza sativa*) leaf with the highest level detected at the grain-filling stage, and it is down-regulated by drought stress conditions. Overexpression of *Ghd2* resulted in significantly reduced drought resistance, while its knockout mutant showed the opposite phenotype. The earlier senescence symptoms and the transcript up-regulation of many senescence-associated genes (SAGs) in *Ghd2*-overexpressing transgenic rice plants under drought stress conditions indicate that *Ghd2* plays essential roles in accelerating drought-induced leaf senescence in rice. Moreover, developmental and dark-induced leaf senescence was accelerated in the *Ghd2*-overexpressing rice and delayed in the *ghd2* mutant. Several SAGs were confirmed to be regulated by Ghd2 using a transient expression system in rice protoplasts. Ghd2 interacted with several regulatory proteins, including OsARID3, OsPURα, and three 14-3-3 proteins. *OsARID3* and *OsPURα* showed expression patterns similar to *Ghd2* in rice leaves, with the highest levels at the grain-filling stage, whereas *OsARID3* and the *14-3-3* genes responded differently to drought stress conditions. These results indicate that *Ghd2* functions as a regulator by integrating environmental signals with the senescence process into a developmental programme through interaction with different proteins.

## Highlight

A novel *CONSTANS*-like gene in rice regulates developmental leaf senescence and confers drought sensitivity through the acceleration of senescence.

## Introduction

Leaf senescence is a precisely controlled process at the final stage of leaf growth and development, during which leaf cells are dismantled and recycled, ultimately leading to cell death (Lim *et al.*, 2007). The initiation of leaf senescence is age dependent under optimal growth conditions, and macromolecules from source leaves are degraded and relocated to support the growth of actively growing tissues such as young leaves and reproducing seeds. Many unfavourable environmental conditions can cause premature senescence, including various abiotic and biotic stresses, among which drought stress constitutes one of the major abiotic factors threatening rice (*Oryza sativa*) yield (Zhang and Zhou, 2013; Albacete *et al.*, 2014).

The most prominent visible change in senescent leaves is chlorophyll breakdown and degradation, and chloroplast dismantling is a major event during senescence (Ougham *et al.*, 2008). At the metabolic level, catabolism takes the place of carbon assimilation. The generation of reactive oxygen species (ROS) is one of the earliest responses under stresses and senescence, and ROS, which primarily affect the chloroplast, are also signalling molecules which trigger senescence (Khanna-Chopra, 2012; Wang *et al.*, 2013; Pinto-Marijuan and Munne-Bosch, 2014). During the senescence process, most of the free amino acid pools increase, and amino acids with a high ratio of nitrogen to carbon (N:C), including asparagine and arginine, can serve as nitrogen storage and transport compounds (Tetley and Thimann, 1974; Hildebrandt *et al.*, 2015; Gaufichon *et al.*, 2016). Sugar accumulation can also trigger leaf senescence (Wingler *et al.*, 2006; Wingler and Roitsch, 2008).

A large number of senescence-related genes (SAGs) are up-regulated during senescence. They are involved in various aspects of the senescence process, including the onset of senescence, the breakdown and remobilization of biomolecules, regulation of the time course, and protection of senescent cells to ensure the accomplishment of the senescence event (Lee *et al.*, 2001; Liu *et al.*, 2011; Li *et al.*, 2014). Among these SAGs, genes involved in the breakdown of chlorophylls such as *SGR* (*stay green*), *NYC1* (*non-yellow coloring1*), *PAO* (*pheide a oxygenase*), and *RCCR* (*chlorophyll catabolite reductase*) are conserved in various species (Hortensteiner and Krautler, 2011; Balazadeh, 2014).

Many stress-responsive genes overlap with SAGs during senescence (Chen, 2002; Guo and Gan, 2012), and some regulatory genes are reported to control both stress and senescence processes. For instance, the *Arabidopsis thaliana* NAC transcription factor ANAC092/AtNAC2/ORE1 is involved in age-dependent senescence control, and disruption of *ANAC092* increases the rate of seed germination under saline conditions (Balazadeh *et al.*, 2010). *NAC016* promotes both senescence and drought stress responses by repressing *AREB1* transcription through a trifurcate feed-forward regulatory loop involving *NAP* (Kim *et al.*, 2013; Sakuraba *et al.*, 2015). The submergence-tolerance gene *SUB1A* enhances rice tolerance to drought and oxidative stress through a delay of leaf senescence (Fukao *et al.*, 2012). A CCCH-type zinc finger family gene *OsTZF1* delays leaf senescence, and improves rice tolerance to high-salt and drought stress conditions (Jan *et al.*, 2013). An S-domain receptor-like kinase OsSIK2 enhances rice tolerance to salt and drought stress conditions, and delays dark-induced senescence (Chen *et al.*, 2013*a*).

The *CONSTANS* (*CO*) gene identified in Arabidopsis plays an important role in the photoperiod pathway of flowering (Putterill *et al.*, 1995). CO contains both CCT and B-box domains, and belongs to the *CO*-like or *BBX* gene family, defined with an emphasis on one of the two domains by different groups (Lagercrantz and Axelsson, 2000; Griffiths *et al.*, 2003; Khanna *et al.*, 2009; Crocco and Botto, 2013; Gangappa and Botto, 2014). Most *CO*-like genes containing a CCT domain are reported to be involved in the control of flowering time in various plant species. In rice, several such genes control the rice heading date, including *Hd1*, *OsCOL3*, *OsCOL4*, *Ghd7*, *DTH2*, *OsCOL10*, *OsCCT01*, *OsCCT11*, and *OsCCT19* (Yano *et al.*, 2000; Kim *et al.*, 2008; Xue *et al.*, 2008; Lee *et al.*, 2010; Wu *et al.*, 2013; Zhang *et al.*, 2015; Tan *et al.*, 2016). To date, only a few members of this family have been identified as regulators of abiotic stress responses. In Arabidopsis, overexpression of *BBX24* (*STO*) and *AtCOL4* improves salt tolerance (Nagaoka and Takano, 2003; Min *et al.*, 2015). Suppression of *BBX24* in *Chrysanthemum* decreases tolerance to freezing and drought stresses, whereas overexpression of this gene enhances tolerance to the stresses (Yang *et al.*, 2014). *Ghd7*, which is a very important *CO*-like gene controlling grain number, plant height, and heading date in rice (Xue *et al.*, 2008), also regulates stress tolerance. The expression of *Ghd7* is up-regulated by cold treatment, but it is repressed by drought, abscisic acid (ABA), and high-temperature treatments (Weng *et al.*, 2014). Overexpression of *Ghd7*^HJ19^ (an allele from rice variety Hejiang19) reduces the drought resistance of rice, and transformants containing an artificial microRNA construct of *Ghd7* in the background of rice variety Zhonghua 11 show increased drought resistance (Weng *et al.*, 2014). However, the regulation mechanism by which Ghd7 participates in stress tolerance is largely unknown.

In this work, we found that the novel *CO*-like gene *Ghd2*, which is a close homologue of *Ghd7* and has similar roles in the control of grain number, heading date, and plant height, positively regulates drought stress-triggered early senescence in rice. Overexpression of *Ghd2* accelerated developmental and dark-induced senescence. Ghd2 activated the expression of many SAGs. Furthermore, Ghd2 was shown to interact with several regulatory proteins, including OsARID3, OsPURα, and three 14-3-3 proteins. Our results suggest that Ghd2 is a multifunctional regulator involved in developmental and drought-induced leaf senescence.

## Materials and methods

### Vector construction and rice transformation

The cDNA sequence of *Ghd2* was amplified from rice variety Zhonghua 11 (ZH11) and cloned into the T-vector (Promega). The cDNA was digested with *Kpn* I and *Bam* HI and cloned into the destination vectors pCAMBIA1301H-HPT (hygromycin resistance and driven by a rice *LEA3* promoter) and pCAMBIA1301U-HPT-flag (hygromycin resistance, driven by a maize *Ubiquitin* promoter, and fused to 3×flag). To construct a CRISPR vector, a spacer sequence was cloned into the *Bsa* I-digested entry vector pOs-sgRNA, and then into the destination vector pH-Ubi-cas9-7 using the Gateway recombination reaction (Invitrogen). The overexpression and CRISPR vectors were introduced into the rice ZH11 cultivar by *Agrobacterium*-mediated transformation (Lin and Zhang, 2005).

### Plant growth and stress treatments

To detect the diurnal expression pattern of *Ghd2* under short day (SD) and long day (LD) conditions, wild type (WT) rice ZH11 were grown in a growth chamber (10h light/14h dark cycle for SD and 14h light/10h dark cycle for LD) and the top-second leaves were sampled at 33 days after germination (DAG).

To detect the transcript levels of *Ghd2* under drought, dark, and ABA treatments, 2-week-old rice plants were subjected to drought stress (placing rice plants on facial tissues), dark (transferring the seedlings to a growth chamber without lighting), and ABA (transferring the seedlings to 100 µM ABA solution) treatments, and the shoots were sampled at the designated time points (0.5, 1, 3, and 6h). Rice plants kept in water were sampled at each time point to serve as controls.

The *Ghd2*-overexpression (*Ghd2*-OE) lines of the T_1_ generation with one copy of a T-DNA insertion (lines 7, 15, 16, 21, and 25) were planted for identification of the segregated non-transgenic (WT') and transgenic genotypes. Seeds from each genotype were harvested separately for further experiments.

For the drought and dark treatments, *Ghd2*-OE seeds were germinated on half-strength Murashige and Skoog (MS) medium (Murashige and Skoog, 1962) containing 50mg/L hygromycin, while the WT' seeds were germinated on half-strength MS medium without hygromycin. At 10 DAG, seedlings were planted in pots with soil. At 30 DAG, drought (no watering until severe leaf rolling) and dark (no light for one week) stresses were applied. The top-second leaves from the WT' and *Ghd2*-OE plants were sampled when they became rolled during drought stress treatment for 3,3′-diaminobenzidine (DAB) staining, H_2_O_2_ quantification, transmission electron microscopy (TEM), scanning electron microscopy (SEM), and gene expression level detection. After 7 days of dark treatment, the leaves were sampled for TEM observation. The WT' and *Ghd2*-OE plants were grown in the field and progressive drought stress started at 60 DAG.

A DNA fragment harbouring the spacer sequence (−49 to +1019 from the start codon) in the segregated non-transgenic *Ghd2*-CRISPR plants was amplified and sequenced to determine the mutation. Seeds with a homozygous mutation near the protospacer adjacent motif were harvested separately. For phenotypic observation at the seedling stage, *Ghd2*-CRISPR and ZH11 seedlings were grown in pots and exposed to drought stress conditions the same as for the *Ghd2*-OE plants. For phenotypic observation at the reproductive stage, seeds of *Ghd2*-CRISPR and ZH11 were grown in the field and polyvinyl chloride (PVC) pots. Moderate drought stress was attained by stopping irrigation to the plants grown in the field at the booting stage until the leaves became fully rolled when observed at noon. Severe drought stress was attained by stopping the watering of the plants grown in PVC pots at the booting stage until the leaves began to turn yellow. The top-second leaves from the ZH11 and *Ghd2*-CRISPR plants during the drought stress treatment were sampled for gene expression level detection.

### Gene expression quantification

The expression level of *Ghd2* at the T_0_ generation was quantified by northern blot analysis using an α-^32^P-dCTP-labelled *Ghd2*-specific probe. The expression levels of other genes were detected by real-time quantitative reverse transcription (qRT)-PCR analysis (Livak and Schmittgen, 2001) using first-strand cDNA synthesized by Superscript III reverse transcriptase (Invitrogen) and performed with FastStart Universal SYBR Green Master (Rox) (Roche) on a real-time PCR system StepOnePlus or QuantStudio 6 Flex (Applied Biosystems). The rice *Ubiquitin* gene was used as the internal control.

### DAB staining and H_2_O_2_ quantification

For DAB staining, leaves were immersed in 0.1mg/mL DAB with 50mM Tris-acetate buffer (pH 3.2), vacuum infiltrated, and placed in the light for 2 days at room temperature. The chlorophylls were removed by placing the samples in 100% ethanol at 37°C before photographing. The content of H_2_O_2_ was quantified using the Amplex® Red Hydrogen Peroxide/Peroxidase Assay Kit (Invitrogen).

### TEM, SEM, and metabolite measurement

Leaves from *Ghd2*-OE and WT' plants at the seedling stage were sampled during drought stress treatment when the leaves became rolled. Normally grown *Ghd2*-OE and WT' plants were sampled at the same time as controls. TEM and SEM were conducted as described previously (Cao *et al.*, 2013). The samples were freeze-dried and metabolites were measured according to the method previously described in detail (Chen *et al.*, 2013*b*). Soluble sugars were determined from freeze-dried samples using the anthrone reagent (Yemm and Willis, 1954).

### Microarray

Three *Ghd2*-OE lines (16, 21, and 25) were grown in pots alongside their respective WT' controls in a half-to-half manner, and the seedlings were harvested for microarray when the leaves became rolled during drought stress treatment. Microarray analysis was conducted by Affymetrix GeneChip service (CapitalBio) following the standard procedure.

### Subcellular localization and bimolecular fluorescence complementation analysis

Ghd2 was fused in-frame with GFP in the vector HBT-sGFP, and Ghd2-GFP and Ghd7-CFP were co-transformed into rice protoplasts isolated from 2-week-old green seedlings using a polyethylene glycol (PEG)-mediated transformation assay. Protoplasts were incubated overnight at room temperature in the dark and the fluorescence signal was observed with a confocal microscope (Leica).

Full-length cDNAs of *Ghd2* and the genes encoding Ghd2-interacting proteins (including *GF14b*, *GF14c*, and *OsARID3*) were amplified with the primers listed in Supplementary Table S1 at *JXB* online. The PCR products were digested and cloned into PvYNE and PvYCE (Waadt *et al.*, 2008), respectively, to produce fusions with N- and C-terminal halves of YFPs, respectively. Combinations of bimolecular fluorescence complementation (BiFC) constructs were co-transformed into rice protoplasts isolated from 2-week-old green seedlings using a PEG-mediated transformation assay. After overnight incubation, the fluorescence was detected by confocal microscopy (Leica).

### Transcriptional activation assay in rice protoplasts

Ghd2 was fused in-frame with the Gal4 DNA-binding domain in the effector vector Gal4BD, and co-transformed with the reporter vector GAL4-LUC. To test the transcriptional activation of the SAGs, Ghd2 was constructed in the effector vector (named ‘None’), while the promoters of the SAGs were constructed in the reporter vector (190LUC). The effector and reporter vectors were co-transformed together with the internal control vector Ubi-Rennila LUC in rice protoplasts isolated from 2-week-old green seedlings by PEG-mediated transformation. The two luciferases which lysed from the overnight incubated protoplasts were incubated with their substrates (Promega) and the luciferase activity was measured using the TECAN Infinite M200 System.

### Transactivation assay in yeast and yeast two-hybrid screening

The full-length and truncated Ghd2 fragments were fused in-frame with the yeast Gal4 DNA-binding domain in the pDEST32 vector (Invitrogen). The fused pDEST32 vectors and the AD502 vector (Invitrogen) were co-transformed into the yeast strain Mav203 (Invitrogen), and the transactivation activity of Ghd2 was indicated by an X-gal assay according to the manufacturer’s manual (Invitrogen). For yeast two-hybrid (Y2H) screening, the Ghd2 deletions (N3 and C3) were used as baits. A cDNA library made from a mixture of rice tissues containing drought-treated leaves, seedlings, and callus was screened on synthetic complete-Leu-Trp-His medium containing 5mM (for N3) and 20mM (for C3) 3-amino-triazol (3-AT).

### *In vivo* pull-down assay

Total proteins were extracted from leaves using extraction buffer [50mM Tris-HCl (pH 7.5), 150mM NaCl, 0.5% NP-40, 1mM phenylmethylsulfonyl fluoride]. Protein extracts were immunoprecipitated with ANTI-FLAG M2 Affinity Gel (A2220, Sigma) overnight at 4°C. Precipitated proteins were suspended with an equal volume of 2× sample buffer [125mM Tris HCl (pH 6.8), 4% SDS, 20% (v/v) glycerol, 0.004% bromophenol blue, 5% 2-mercaptoethanol], denatured at 98°C for 5min, and subjected to SDS-PAGE. The proteins were electroblotted onto polyvinylidene difluoride membrane and an antibody against OsARID3 (Xu *et al.*, 2015) was used for the immunoblot analysis. The chemiluminescence detection kit ImmunStar WesternC (Bio-Rad) was used for the visualization of peroxidase activity of the secondary antibodies.

## Results

### Overexpression of *OsK*/*Ghd2* in rice increased drought sensitivity

**Fig. 1.**
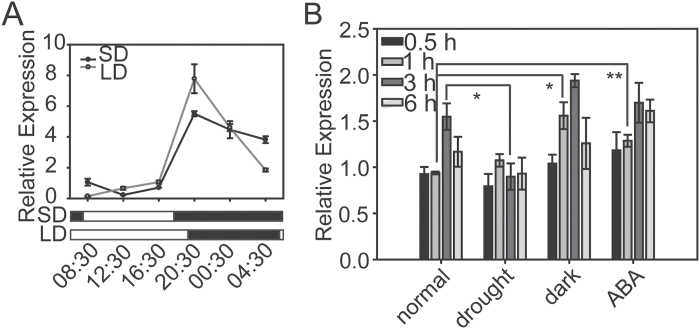
Diurnal expression pattern and the response of the *Ghd2* or *OsK* gene to drought, dark, and ABA treatments. (**A**) Diurnal expression pattern of *Ghd2* under LD and SD conditions. (**B**) Expression analysis of *Ghd2* under drought, dark, and ABA treatments at 0.5, 1, 3, and 6h. Two-week-old seedlings kept in water were sampled simultaneously at each time point to serve as controls. Error bars indicate the SE based on three biological replicates. *P < 0.05, *t*-test; **P < 0.01, *t*-test.

To investigate whether CCT domain-containing genes are involved in the regulation of the drought response in rice, first we checked the expression levels of this family under drought stress conditions. In the published microarray data GSE6901, which contains the gene expression profiles of rice seedlings under drought, salinity, and cold stresses (Jain *et al.*, 2007), we found that three close homologues of *CO*-like genes in group II, originally named *OsJ* (*OsCOL10*), *OsK*, and *OsL* (Griffiths *et al.*, 2003), were down-regulated under drought stress treatment (Supplementary Table S2). In our previous microarray results consisting of global genome expression analyses in shoots, flag leaves, and panicles under drought and salinity stresses (Zhou *et al.*, 2007), we also found that *OsK* (LOC_Os02g49880) was significantly down-regulated under drought stress conditions (Supplementary Table S3). The expression of *OsK* was further investigated under drought, dark, and ABA treatments by performing qRT-PCR. The expression of *OsK* was analysed in the treated rice seedlings with their respective non-treated controls at each time point during the treatment, given that *OsK* exhibited a diurnal expression pattern under both the SD and LD growth conditions (Fig. 1A). Similar to the microarray results, the expression level of *OsK* was significantly down-regulated at 3h of drought stress treatment. However, *OsK* was slightly induced at 1h of dark and ABA treatments (Fig. 1B).

We thereafter overexpressed *OsK* in the rice variety Zhonghua11 using a *LEAP* promoter, which exhibits a moderate expression level under normal growth conditions but it is strongly induced by drought stress and ABA treatment (Xiao *et al.*, 2007). Northern blot was used to identify the overexpressing T_0_ seedlings, and six of them were found to be overexpressing *OsK*(Supplementary Fig. S1A). Southern blot results suggested that five seedling lines (7, 15, 16, 21, and 25) each had one copy of the T-DNA insertion (Supplementary Fig. S1B). Segregated non-transgenic lines (WT′) from the single-copy lines were used as negative controls in the following analyses. Under normal growth conditions, the *OsK*-overexpressing plants showed significantly increased grain number per panicle, plant height, and heading date (Supplementary Fig. S2), which is very similar to the phenotypes of the *Ghd7*-overexpressing rice (Xue *et al.*, 2008). Taking into consideration the commonly adopted rule of gene nomenclature in rice (McCouch, 2008), we renamed *OsK* as *Ghd2*, because this gene confers functions similar to *Ghd7* in the control of **g**rain number, **p**lant height, and heading **d**ate, and it is a close homologue of *Ghd7*.

**Fig. 2.**
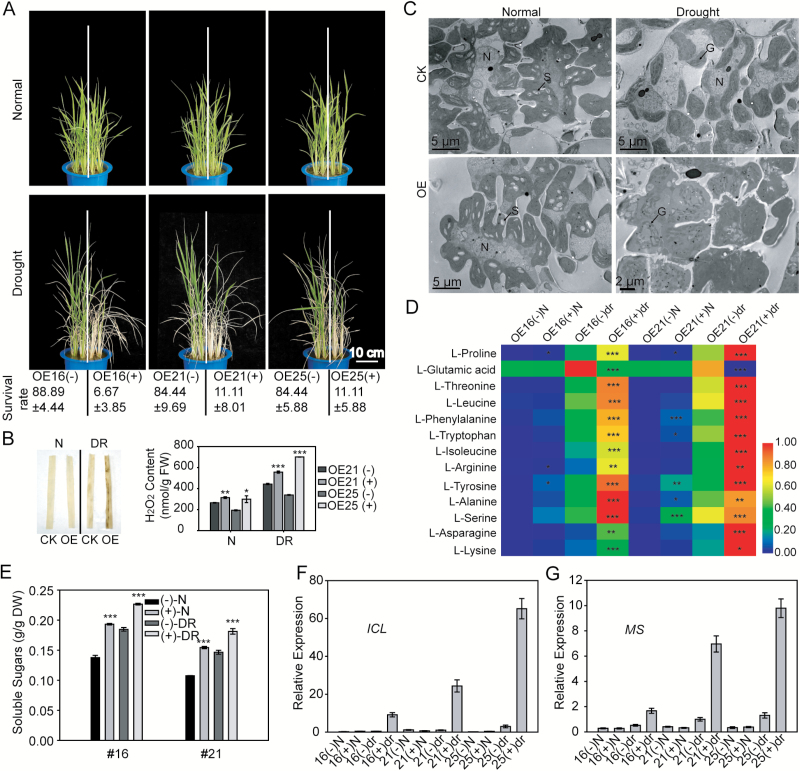
Overexpression of *Ghd2* accelerated drought-induced leaf senescence at the seedling stage. (**A**) Phenotypes of the *Ghd2*-OE and WT' plants under drought stress treatment. Survival rates are shown below. Data represent the mean ± SE based on three replicates. Images presented above show five seedlings grown in one-half of each pot, whereas 15 seedlings grown in one-half of each pot were used for determining the survival rate. ‘+’ and ‘-’ refers to segregated transgenic and non-transgenic plants, respectively, from the *Ghd2*-OE lines. (**B**) H_2_O_2_ content in the *Ghd2*-OE and WT' plant leaves under normal and drought stress conditions. Left, DAB staining; right, H_2_O_2_ quantification. CK, the non-transgenic control (WT'). N, normal; DR, drought treatment; FW, fresh weight. Data represent the mean ± SE (*n* = 3). *P < 0.05, *t*-test; **P < 0.01, *t*-test; ***P < 0.005, *t*-test. (**C**) TEM images of the ultrastructure of *Ghd2*-OE and WT' plant leaves under normal and drought stress conditions. G, grana stack; N, nucleus; S, starch granule. (**D**) Contents of free amino acids in *Ghd2*-OE and WT’ plants at the seedling stage under normal and drought stress conditions. The data were analysed using the Heatmap Illustrator (http://hemi.biocuckoo.org/down.php). The data represent the mean ± SE (*n* = 3). *P < 0.05, *t*-test; **P < 0.01, *t*-test; ***P < 0.005, *t*-test. (**E**) Contents of soluble sugars in the *Ghd2*-OE and WT' plants. DW, dry weight. ***P < 0.005, *t*-test. (**F** and **G**) Validation of the expression changes of *ICL* (F) and *MS* (G) in a microarray analysis using qRT-PCR.

To check whether *Ghd2* overexpression affected the drought resistance of rice, the *Ghd2*-OE and WT' plants were grown in the same pot in a half-to-half manner, and drought stress was applied at the seedling stage. To our surprise, the *Ghd2*-OE plants were more sensitive to drought stress than the WT'. During drought stress treatment, the *Ghd2*-OE plants showed an earlier leaf-rolling phenotype and less than 12% of them survived after recovery from a moderate drought stress treatment, whereas the survival rates for the WT' plants were above 84% (Fig. 2A). The *Ghd2*-OE plants also showed increased sensitivity to drought stress treatment when grown in the paddy field (Supplementary Fig. S3A). These results suggest that *Ghd2* may negatively regulate drought resistance.

### Drought-induced senescence was accelerated in *Ghd2*-OE plants

We first checked stomata of the *Ghd2*-OE and WT' plants because stomata affect the water-holding capacity of plants during drought stress. The results showed that there was no significant difference between the *Ghd2*-OE and WT' plants in stomatal density under both normal and drought conditions (Supplementary Fig. S4C). The percentage of opening stomata showed no significant difference either (Supplementary Fig. S4D). The water loss rates of detached rice leaves from the *Ghd2*-OE plants were similar to those of WT' plants (Supplementary Fig. S4E). Therefore, the role of *Ghd2* in promoting drought sensitivity may not be related to stomata.

Careful observation of the phenotypic changes of the *Ghd2*-OE plants during the drought stress treatment revealed an early senescence symptom. As shown in Supplementary Fig. S3A (left), after being stressed by the progressive drought stress treatment (before leaf rolling), the leaves of the *Ghd2*-OE plants, especially in the leaf tips, began to turn yellow, but the leaves in the WT' plants remained green, similar to the non-treated rice plants. We performed DAB staining and found that H_2_O_2_ accumulated much more in the *Ghd2*-OE plants than in the WT' plants under drought stress treatment (Fig. 2B left panel). We also performed H_2_O_2_ quantification using an Amplex Red assay. A significant difference was detected under drought stress conditions, and the difference was detected even in leaves of normally grown plants, although the content was relatively low (Fig. 2B right panel).

Previous studies suggest that, as senescence proceeds, the ultrastructure of the leaf cells undergoes typical changes, as follows: chloroplasts become swelled and round, grana stacks and intergrana become disordered, and chloroplasts finally shrink and chloroplast components are completely decomposed (Kusaba *et al.*, 2007; Park *et al.*, 2007). We examined the ultrastructure of the *Ghd2*-OE and WT' leaves using TEM. As shown in Fig. 2C, there were no obvious differences under normal growth conditions, but under drought stress conditions, the chloroplasts in the *Ghd2*-OE plants were obviously swelled and the array of grana stacks were slightly disordered. However, the WT' chloroplasts maintained an appearance similar to the chloroplasts of normally grown plants, with the exception of the exhausted starch granules. These observations indicate that the drought-induced senescence was accelerated in the *Ghd2*-OE leaves compared to the WT'.

It is known that most of the free amino acid pools increase during the senescence process. We used gas chromatography-mass spectrometry to determine the contents of amino acids. The results showed that the contents of most of the free amino acids were significantly higher in the *Ghd2*-OE plants than in the WT' under drought stress conditions (Fig. 2D). We also measured soluble sugar content and the result showed that soluble sugars accumulated significantly more in the *Ghd2*-OE plants than in the WT' under both normal and drought stress conditions (Fig. 2E).

The above results indicate that *Ghd2* overexpression may accelerate drought-induced leaf senescence. To further confirm this at the gene expression level, we compared the whole genome expression profiles to identify differentially expressed genes (DEGs) between the *Ghd2*-OE and WT' plants under drought stress treatment. Leaves from three *Ghd2*-OE lines (line numbers 16, 21, and 25) and their corresponding WT's were sampled for microarray analysis when the leaves became rolled during the drought stress treatment. Gene Ontology (GO) analysis revealed that ‘amino acid transmembrane transport’ and ‘anion transmembrane transport’ were the main overrepresented terms under the ‘biological processes’ category. The GO terms with the highest proportion of DEGs under the ‘molecular function’ category were ‘transmembrane transporter activity’ and ‘symporter activity’ (Supplementary Table S4). Many SAGs showed higher expression levels in the *Ghd2*-OE plants than in the WT' (Table 1). Of these up-regulated SAGs, genes involved in chlorophyll degradation, including *OsNAP*, *OsSGR*, *OsNYC3*, *OsPAO*, *OsRCCR1*, and *OsRCCR3*, showed more than 2-fold higher expression in the *Ghd2*-OE plants. Three genes (*OsPPDKB*, *OsAS1*, and *OsGS1;2*), which function in asparagine synthesis for the long-distance transport of nitrogen (Funayama *et al.*, 2013; Hartmann *et al.*, 2015; Ohashi *et al.*, 2015) were also up-regulated in the *Ghd2*-OE plants. Notably, the expression of two key genes in the glyoxylate cycle, *Isocitrate lyase* (*ICL*) and *Malate synthase* (*MS*), were elevated to very high levels in the *Ghd2*-OE plants under drought stress conditions. The expression levels of *ICL* and *MS* were also increased in the WT' plants during drought stress treatment, but the induction was significantly stronger in the *Ghd2*-OE plants (Fig. 2F, G). The glyoxylate cycle is involved in the oil reserve mobilization in the germination of oil crops, and the activation of the glyoxysome during leaf senescence is proposed to mobilize thylakoid membrane lipids (Eastmond and Graham, 2001; Graham, 2008). Therefore, the up-regulation of key genes of the glyoxylate cycle in the *Ghd2*-OE plants may imply a faster mobilization of the thylakoid membrane lipids.

**Table 1.** Microarray results of SAGs in the *Ghd2*-OE and WT’ plants during drought stress treatment.

ID	Name	#16 (+/-)	#21 (+/-)	#25 (+/-)
LOC_Os07g34520	*ICL*	40.61	22.73	23.10
LOC_Os04g40990	*MS*	14.42	8.55	8.35
LOC_Os09g36200	*SGR*	2.11	3.08	1.78
LOC_Os03g18130	*AS1*	1.82	2.10	2.78
LOC_Os03g12290	*GS1;2*	1.48	3.02	2.27
LOC_Os10g25030	*RCCR1*	2.66	2.85	2.04
LOC_Os03g21060	*NAP*	1.45	2.79	2.02
LOC_Os10g25030	*RCCR3*	2.66	2.78	1.95
LOC_Os05g33570	*PPDKB*	1.30	2.72	1.40
LOC_Os06g24730	*NYC3*	2.05	2.67	2.18
LOC_Os03g05310	*PAO*	1.52	2.09	1.46
LOC_Os10g25040	*RCCR2*	1.36	1.77	1.92
LOC_Os07g37250	*NYC4*	1.22	1.58	1.30
LOC_Os02g57260	*l57*	1.31	1.45	1.38
LOC_Os01g12710	*NYC1*	1.35	1.44	1.71

‘+/-’, ratios of expression levels in transgenic positive plants versus expression levels in transgenic negative plants.

Our findings with regards the ultrastructure of the chloroplast; H_2_O_2_, free amino acid, and soluble sugar content; and the gene expression of SAGs together suggest that the senescence process is accelerated by *Ghd2* overexpression.

### Disruption of *Ghd2* enhanced drought resistance and delayed leaf senescence

**Fig. 3.**
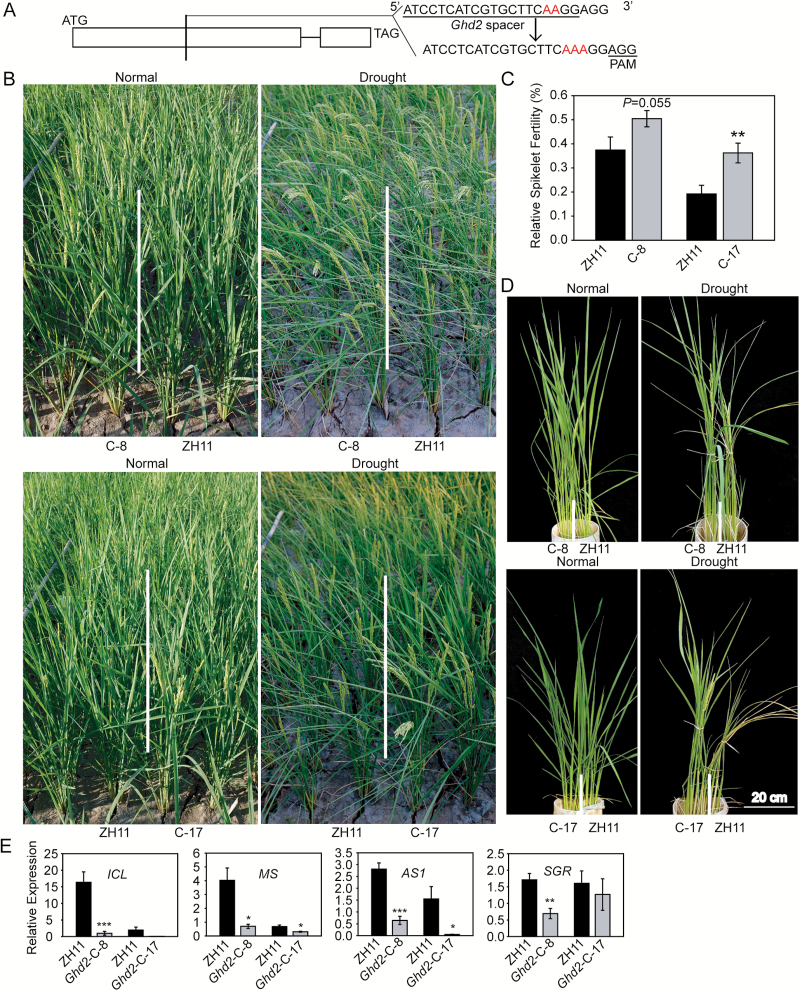
*Ghd2*-CRISPR plants showed delayed drought-induced leaf senescence. (**A**) Diagram showing the structure of *Ghd2* for CRISPR. (**B**) Phenotypes of the *Ghd2*-CRISPR (C-x) and WT (ZH11) plants during drought stress treatment in the field facilitated with a moveable rain-off shelter. (**C**) Relative spikelet fertility of the *Ghd2*-CRISPR and WT plants after moderate drought stress treatment at the panicle development stage in the field. Data represent the mean ± SE (*n* = 12). **P < 0.01, *t*-test. (**D**) Phenotypes of the *Ghd2*-CRISPR and WT plants during severe drought stress treatment at the reproductive stage in PVC pots. (**E**) Real-time qRT-PCR results showing the expression levels of the SAGs in the *Ghd2*-CRISPR and WT plants during drought stress treatment. Data represent the mean ± SE (*n* = 4). *P < 0.05, *t*-test; **P < 0.01, *t*-test; ***P < 0.005, *t*-test.

We further checked whether knockout mutation of *Ghd2* could affect drought resistance. CRISPR/CAS9 technology (Miao *et al.*, 2013) was used to produce a mutant of *Ghd2* because a T-DNA insertion mutant for this gene was unavailable. We designed sgRNA targeted to the middle of the first exon of the *Ghd2* gene (Fig. 3A), and the CRISPR construct was transformed into ZH11. Single-copy transgenic lines were identified by Southern blot analysis (Supplementary Fig. S1C), and progenies without CAS9 and the hygromycin-resistance gene (*Hpt*) insertion were selected by PCR using primers for both the CAS9 and *Hpt* sequences. Sequencing of the PCR amplifications covering the target site suggested that the homozygous lines (line numbers 8, 17, and 20) were mutated by a single nucleotide (A) insertion.

At the seedling stage, the *Ghd2*-CRISPR lines showed improved drought resistance (Supplementary Fig. S3B). During a moderate drought stress treatment at the panicle development stage, the *Ghd2*-CRISPR lines showed an obvious delay of leaf-rolling compared with the WT (Fig. 3B). The relative spikelet fertility was higher in the *Ghd2*-CRISPR plants (Fig. 3C). We also tested leaf senescence of the *Ghd2*-CRISPR plants grown in PVC pots with severe drought stress treatment at the reproductive stage. With the extended period of drought stress, the older leaves of the WT plants began to turn yellow, whereas all of the *Ghd2*-CRISPR leaves remained green, indicating that the drought-induced senescence was delayed in the *Ghd2*-CRISPR plants (Fig. 3D). The H_2_O_2_ content was also significantly lower in the *Ghd2*-CRISPR plants under the drought stress conditions (Supplementary Fig. S5). qRT-PCR showed that the SAGs, including *ICL*, *MS*, *OsAS1*, and *OsSGR*, were down-regulated in the *Ghd2*-CRISPR plants during drought stress treatment (Fig. 3E). These results further suggest that *Ghd2* positively affects drought-induced leaf senescence.

### *Ghd2* regulates developmental and dark-induced senescence

**Fig. 4.**
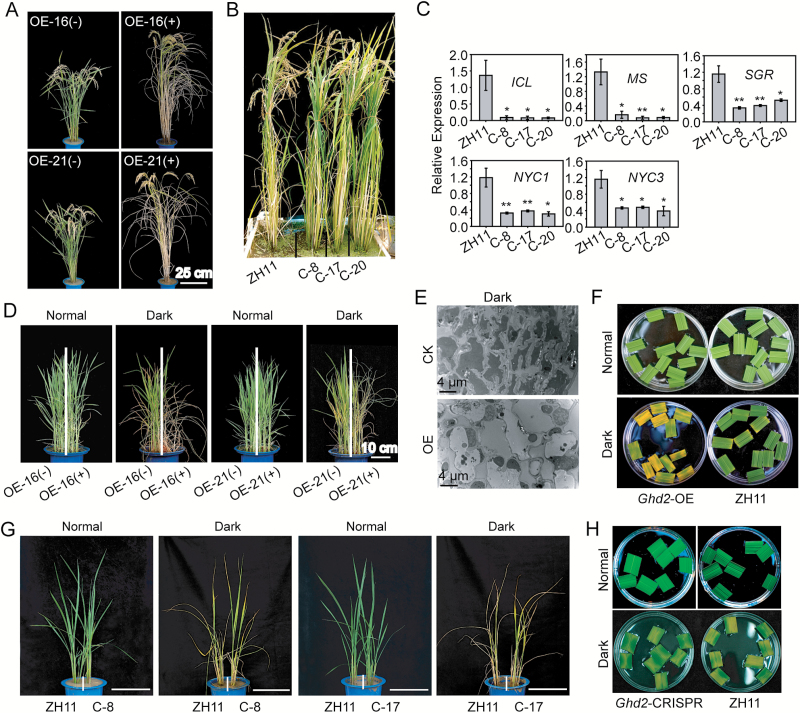
Phenotypes of the *Ghd2*-OE and *Ghd2*-CRISPR plants during developmental and dark-induced leaf senescence. (**A**) Phenotypes of the *Ghd2*-OE and WT’ rice at 35 DAF. (**B**) Phenotypes of the *Ghd2*-CRISPR and WT rice at 50 DAF. (**C**) Expression of SAGs in the leaves of *Ghd2*-CRISPR and WT plants at the late grain-filling stage. Data represent the mean ± SE (*n* = 4). *P < 0.05, *t*-test; **P < 0.01, *t*-test. (**D**) Phenotypes of the *Ghd2*-OE(+) and WT' (*Ghd2*-OE(-)) rice seedlings after dark treatment. ‘+’ and ‘-’ refers to segregated transgenic and non-transgenic plants, respectively, from the *Ghd2*-OE lines. (**E**) TEM images of the ultrastructure of *Ghd2*-OE and CK (the non-transgenic control, WT') plant leaves after dark treatment. (**F**) Phenotypes of the *Ghd2*-OE and WT plant leaves after dark treatment. Segments of top-second leaves were incubated in water and dark-treated for 3 days before photographing. (**G**) Phenotypes of the *Ghd2*-CRISPR and WT rice seedlings after dark treatment. Bar = 20cm. (**H**) Phenotypes of the *Ghd2*-CRISPR and WT plant leaves after dark treatment. Leaf segments were dark-treated for 1 week.

When grown in the field under non-stressed conditions, *Ghd2*-OE plants showed mild early senescence symptoms before flowering (Supplementary Fig. S2A), and the symptoms became increasingly obvious during the grain-filling process. At 35 days after flowering (DAF), the *Ghd2*-OE plants exhibited a significantly accelerated leaf-yellowing phenotype (Fig. 4A). In contrast, leaf senescence was delayed in the *Ghd2*-CRISPR plants at the late grain-filling stage, and the *Ghd2*-CRISPR plants exhibited a significantly delayed leaf-yellowing phenotype at 50 DAF (Fig. 4B). We also examined the expression of the SAGs at the late grain-filling stage, and found that *ICL*, *MS*, *OsSGR*, *OsNYC1*, and *OsNYC3* had relatively lower expression levels in the *Ghd2*-CRISPR plants than in the WT (Fig. 4C). Of note, *Ghd2* was strongly expressed in the matured leaves approaching senescence, especially at the grain-filling stage (Supplementary Fig. S6). These results suggest that *Ghd2* may also be involved in the regulation of developmental leaf senescence, especially at the grain-filling stage.

We further examined whether dark treatment could accelerate the senescence of *Ghd2*-OE plants because dark treatment has frequently been used to simulate synchronous senescence (Cha *et al.*, 2002; Kusaba *et al.*, 2007; Morita *et al.*, 2009). *Ghd2*-OE and WT' seedlings grown in the same pots were exposed to dark conditions for 1 week and then recovered with normal light conditions. As expected, the *Ghd2*-OE plants showed accelerated leaf senescence with dark treatment, with leaves yellowing faster and more seedlings dying after recovery (Fig. 4D). After dark treatment, the number of thylakoids was significantly lower in the *Ghd2*-OE leaf blade tips than in WT', as observed by TEM (Fig. 4E). The leaf segments of *Ghd2*-OE showed faster yellowing than the WT' after the dark treatment (Fig. 4F). In contrast to the *Ghd2*-OE plants, both the intact (Fig. 4G) and detached (Fig. 4H) leaves from the *Ghd2*-CRISPR plants showed delayed senescence compared to WT under dark treatment. The above results together suggest that *Ghd2* promotes both developmental and dark-induced senescence in addition to drought-induced senescence.

### Ghd2 activated the expression of senescence-related genes

**Fig. 5.**
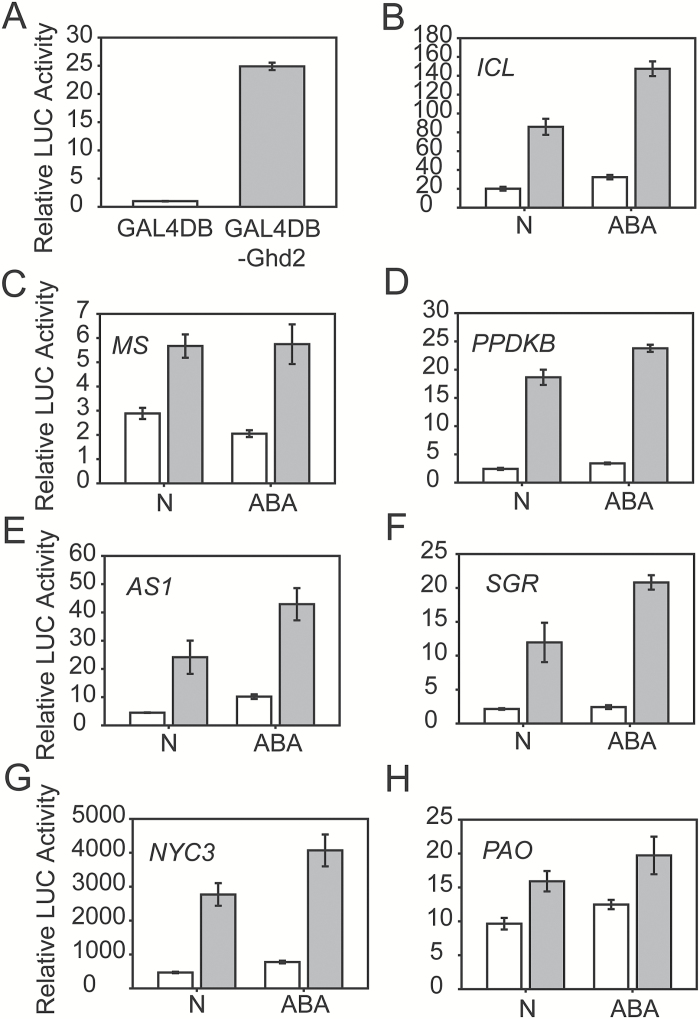
Ghd2 activated the expression of SAGs in protoplasts. Effector (Ghd2) and reporter (promoter) constructs were co-transformed into rice protoplasts using a dual firefly-Renilla luciferase system. (**A**) Trans-activation activity of Ghd2 in rice protoplasts. 35S::Gal4BD:Ghd2 was co-transformed with Gal4::LUC and strongly induced LUC expression. (**B**–**H**) 35S::Ghd2 induced the expression of LUC driven by the promoters of SAGs under normal and ABA treatment conditions. The white bar shows the LUC activity of the empty effector vector and the grey bar the LUC activity of 35S::Ghd2 co-transformed with the reporter constructs.

Similar to other reported CO-like proteins, a Ghd2-GFP fusion was localized in the nuclei of rice protoplasts (Supplementary Fig. S7A). It has been reported that CO-like proteins possess trans-activation activity in yeast, and the activation domain is located in the middle region between the CCT domain and the B-box domain (Ben-Naim *et al.*, 2006; Min *et al.*, 2015). Ghd2 also possessed trans-activation activity, and the activation assay of a series of deletion mutants of Ghd2 in yeast showed that the activation domain was also located in the middle region of the Ghd2 protein (Supplementary Fig. S7). We also confirmed that Ghd2 has high trans-activation activity in protoplasts (Fig. 5A).

Thereafter, we examined whether Ghd2 could activate the expression of some SAGs by using a dual firefly-Renilla luciferase system in rice protoplasts. The results showed that overexpression of *Ghd2* in rice protoplasts resulted in the up-regulated expression of several SAGs, including *ICL* and *MS* in the glyoxylate cycle, *OsPPDKB* and *OsAS1* in amino acid remobilization, and *OsSGR*, *OsNYC3*, and *OsPAO* in chloroplast degradation (Fig. 5B–H). Because ABA is an important phytohormone participating in leaf senescence (Liang *et al.*, 2014), and *Ghd2* was induced by ABA (Fig. 1B), we treated protoplasts with ABA and observed that ABA treatment enhanced the induction of these SAGs, with the exception of *MS* (Fig. 5B–H). These results indicate that Ghd2 can activate the expression of some SAGs, which partially explains the accelerated senescence by *Ghd2* overexpression.

### Identification of Ghd2-interacting proteins

**Fig. 6.**
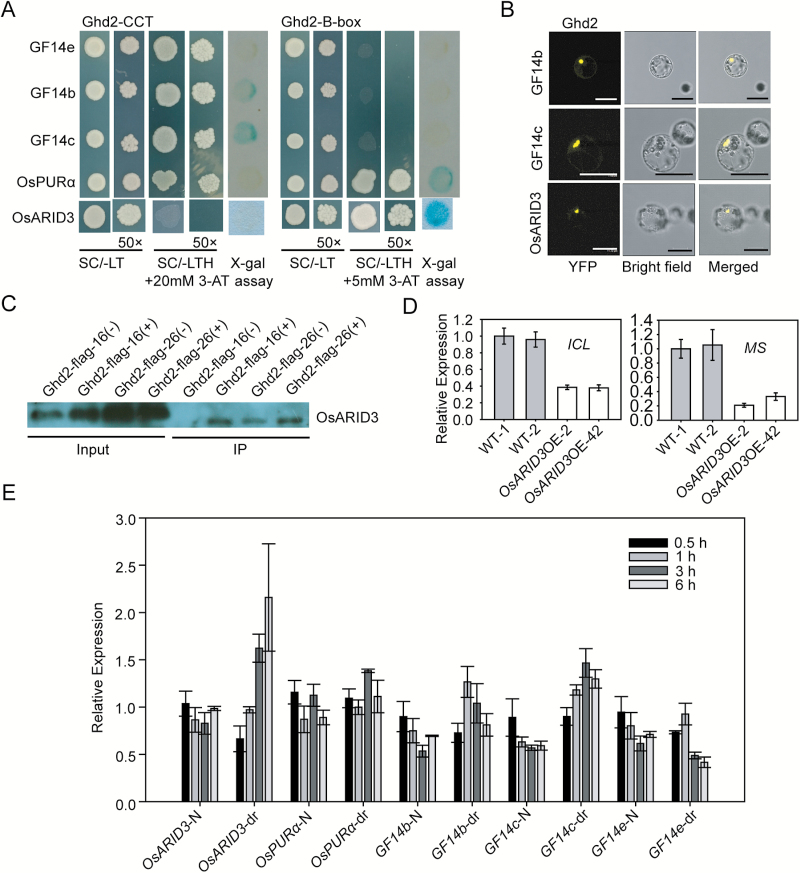
Identification of Ghd2-interacting proteins. (**A**) 14-3-3 proteins (GF14b, GF14c, and GF14e), OsPURα, and OsARID3 interact with C3 or N3 deletions of Ghd2 in Y2H assays. Colonies grown on Synthetic complete-Leu-Trp medium (SC/-LT) were tested by both X-gal assay and selection on Synthetic complete-Leu-Trp-His medium (SC/-LTH) containing 5 and 20mM 3-AT for N3 and C3, respectively. (**B**) 14-3-3 proteins (GF14b and GF14c) and OsARID3 were confirmed to interact with Ghd2 by BiFC. YFP, yellow fluorescent protein. Bar = 20 μm. (**C**) Confirmation of the interaction between Ghd2 and OsARID3 by an *in vivo* pull-down assay. (**D**) Expression of *ICL* and *MS* in the *OsARID3*-OE and WT plants. (**E**) Expression analysis of *OsPURα*, *OsARID3*, and *14-3-3* proteins under drought stress conditions. This figure is available in colour at *JXB* online.

Both the CCT domain and B-box domain are able to mediate protein–protein interactions (Ben-Naim *et al.*, 2006). We performed Y2H screening to identify the interacting proteins of Ghd2. Because the complete Ghd2 protein has strong activation activity, we used N3 and C3, two deletions without self-activation activity and containing N-terminal B-box and C-terminal CCT domains, respectively (Supplementary Fig. S7B), as baits in the screening. Sequencing of the positive clones from the Y2H screening resulted in two candidates (OsARID3 and OsPURα) for N3-interacting proteins and three candidates (OsGF14 b, c, and e) for C3-interacting proteins (Fig. 6A). These candidate proteins were validated to interact with Ghd2 by both the HIS3 and lacZ reporters, except OsGF14e, which could only be validated by the HIS3 reporter. We further confirmed the interactions for OsGF14b, OsGF14c, and OsARID3 by BiFC (Fig. 6B). The interaction between Ghd2 and OsARID3 was further confirmed using an *in vivo* pull-down assay (Fig. 6C).

We checked the microarray results for *OsARID3*-overexpression plants (*OsARID3*-OE) generated previously in our group (Xu *et al.*, 2015). Interestingly, we found that *ICL* and *MS* were down-regulated in the *OsARID3*-OE plants, which is in contrast to the up-regulation of *ICL* and *MS* in the *Ghd2*-OE plants. qRT-PCR analysis confirmed this result (Fig. 6D), suggesting that Ghd2 and OsARID3 may work antagonistically in regulating the two SAGs.

The transcript levels of these Ghd2-interacting proteins were examined in response to drought stress treatment. The results showed that *OsARID3* was induced by drought stress treatment (Fig. 6E), which is the opposite to the drought-suppressed expression of *Ghd2* (Fig. 1B). *OsPURα* showed no significant change in response to drought stress treatment, while the three 14-3-3 genes responded differently, with *GF14b* and *GF14c* being induced and *GF14e* being repressed by drought stress treatment (Fig. 6E). We also checked the diurnal expression patterns of these genes at different developmental stages. We found that the expression patterns of *OsARID3* and *OsPURα* were very similar to *Ghd2*, with the highest levels occurring during the night at the grain-filling stage (Supplementary Fig. S6). These results indicate that Ghd2-interacting proteins may be involved in different biological processes, including developmental and drought-induced leaf senescence regulated by *Ghd2*.

## Discussion

### Different roles of *Ghd2* under optimal growth and stress conditions

*CO*-like genes have been intensively studied with a focus on their roles in photoperiodic flowering, but very limited information related to abiotic stresses has been reported for this gene family. We found that *Ghd2* not only controls grain number, heading date, and plant height (Supplementary Fig. S2A), but also confers drought sensitivity by accelerating drought-induced premature senescence. To date, the molecular link between yield potential and stress tolerance has seldom been addressed. A previous study reported that *Ghd7*, a close homologue of *Ghd2*, plays a negative role in drought resistance (Weng *et al.*, 2014), but the mechanism was not completely revealed. Both *Ghd2* and *Ghd7* are down-regulated by drought stress conditions. Therefore, these *CO*-like genes, including *Ghd2* and *Ghd7*, may be good candidates to investigate the regulation mechanisms of how plants achieve high yield under favourable growth conditions, and how plants avoid severe yield loss or failure of reproduction under unfavourable environmental conditions. *Ghd2* is rhythmically expressed in rice leaves (Fig. 1A) and delays the rice heading date (Supplementary Fig. S2A). The longer vegetative growth period enables rice plants to produce more leaves, and the relatively high expression of *Ghd2* at the grain-filling stage could promote the translocation of nutrients from senescent leaves synthesized during the vegetative phase to developing seeds. On the other hand, down-regulation of *Ghd2* during drought stress conditions alleviates drought-induced leaf senescence, and thus more photosynthetic leaves remain for seed development.

### *Ghd2* confers drought sensitivity by accelerating drought-induced leaf senescence

Senescence is a tightly regulated process accompanied by co-ordinated expression of SAGs. To date, several genes have been characterized to play key roles in the regulation of leaf senescence in rice, especially in processes of chlorophyll breakdown and degradation, including *OsSGR*, *OsNYC1*, *OsNYC3*, *OsPAO*, and *OsRCCR* (Cha *et al.*, 2002; Kusaba *et al.*, 2007; Park *et al.*, 2007; Morita *et al.*, 2009; Tang *et al.*, 2011), which have been used as marker genes for leaf senescence. *OsNAP* has been identified as an important regulator in senescence that directly or indirectly regulates SAGs, and it has been suggested as an ideal marker for the onset of leaf senescence in rice (Liang *et al.*, 2014). Frequently used marker genes for leaf senescence also include *Osl57* and *Osl85*, which are involved in fatty acid degradation and remobilization and encode 3-ketoacyl CoA thiolase and ICL respectively (Lee *et al.*, 2001). In this study, we found most of these known marker genes were up-regulated in the *Ghd2*-OE plants during drought stress (Table 1), suggesting that Ghd2 may regulate leaf senescence by altering the expression of these genes.

Leaf senescence is associated with nutrient remobilization, especially the recycling of nitrogen from the photosynthetic apparatus. Amino acid metabolism is reprogrammed during the senescence process and Asn in particular can serve as the major shunt for storage and long-distance transport of nitrogen from sources to sinks because of its high N:C ratio and relatively inert feature (Lam *et al.*, 1994; Lam *et al.*, 2003). The levels of most free amino acids including Asn were significantly higher in the *Ghd2*-OE plants than in the WT′ under drought stress conditions, further supporting the roles of Ghd2 in accelerating senescence. Expression of key genes in the glyoxylate cycle, *ICL* and *MS*, are increased during leaf senescence (Pastori and Rio, 1997), and PPDK was suggested to salvage carbon from glyoxysome- mediated lipid degradation, thus providing the precursor for the synthesis of Asn (Lin and Wu, 2004; Hartmann *et al.*, 2015). Rice OsAS1 is responsible for the final step in Asn biosynthesis and OsGS1;2 is involved in the pathway (Ohashi *et al.*, 2015). The up-regulation of *ICL*, *MS*, *OsPPDKB*, and *OsAS1* in the *Ghd2*-OE plants during drought stress (Table 1) and the activation of these genes by Ghd2 in rice protoplast (Fig. 5) are in accordance with the higher Asn content (Fig. 2D). These results suggest that Ghd2 may reprogram amino acid metabolism through regulation of genes such as *ICL*, *MS*, *OsPPDKB*, and *OsAS1* to redirect C and N transport from sources to sinks. Given that the glyoxylate cycle can convert membrane lipids to soluble carbohydrates or substrates for energy metabolism (Eastmond and Graham, 2001), we speculate that the carbohydrates salvaged from the glyoxylate cycle can also partly contribute to the higher soluble sugar content in the *Ghd2*-OE plants (Fig. 2E). Furthermore, the term ‘transmembrane transport’ was overrepresented in the up-regulated genes in the *Ghd2*-OE plants (Supplementary Table S4), implying a possible relevance to accelerated nutrient transport. This evidence together suggests that Ghd2 positively regulates the expression of SAGs during drought stress to accelerate senescence-related processes such as chloroplast breakdown and degradation, metabolism reprogramming, and nutrient mobilization, which finally leads to drought sensitivity.

### Ghd2 promotes developmental and dark-induced senescence

Under optimal growth conditions, leaf senescence is initiated in an age-dependent manner, and crops including rice undergo leaf senescence at the grain-filling and maturation stages in the field (Lim *et al.*, 2007; Schippers, 2015). Analysis of *OsNAP*, which regulates leaf senescence in an age-dependent manner and indicates the onset of senescence, suggests that the age-dependent leaf senescence process in rice is initiated at the tillering stage but not before the four-leaf stage (Liang *et al.*, 2014). In line with this result, the *Ghd2*-OE plants showed slightly accelerated leaf senescence at the tillering stage in the field (Supplementary Fig. S2A), and senescence was significantly accelerated during the grain-filling and maturation stages (Fig. 4A), whereas senescence was delayed in the *Ghd2*-CRISPR plants (Fig. 4B), suggesting that *Ghd2* can also promote developmental senescence in addition to drought-induced senescence. Because developmental leaf senescence at the grain-filling and maturation stages involves nutrient translocation from senescent leaves to developing seeds (Liang *et al.*, 2014), the relatively high expression of *Ghd2* in matured leaves especially at the grain-filling stage (Supplementary Fig. S6) could be related to nutrient translocation in the developmental senescence process. The acceleration of leaf senescence at the grain-filling stage by *Ghd2* is reasonable because *Ghd2* overexpression could delay flowering time (Supplementary Fig. S2) and there is a great demand for the late flowering plants to quickly finish the grain-filling process before encountering unfavourable low temperature conditions. In addition, accelerated dark-induced leaf senescence in *Ghd2*-OE plants and delayed dark-induced leaf senescence in *Ghd2*-CRISPR plants (Fig. 4D–H) further confirms the involvement of *Ghd2* in senescence regulation.

### Ghd2-interacting proteins and their diverse roles

It has been reported that co-factors such as HAP3 and HAP5 could interact with CO-like proteins to form a complex and bind target genes (Ben-Naim *et al.*, 2006; Wenkel *et al.*, 2006). In addition to HAPs, other CO-like interacting proteins could also play important roles in the regulation of *CO*-like genes. 14-3-3 proteins *μ* and *ν* interact with CO and influence various biological processes, such as transition to flowering, early phytochrome response, red light signalling, and chloroplast development (Folta *et al.*, 2008; Mayfield *et al.*, 2007; Mayfield *et al.*, 2012). Specifically, 14-3-3 proteins form a florigen activation complex with Hd3a and OsFD1 to control flowering time (Taoka *et al.*, 2011). Some 14-3-3 genes have been reported to be responsive to various stresses, such as drought, heat, salt, and cold (Chen *et al.*, 2006; Denison *et al.*, 2011). In this study, we have identified three close homologues of the 14-3-3 protein, including GF14b, GF14c, and GF14e, which specifically interact with the CCT domain of Ghd2. We found that the three rice 14-3-3 genes responded differently to drought stress conditions, with *GF14b* and *GF14c* being induced and *GF14e* being repressed by drought stress treatment. The wide spread participation of 14-3-3 proteins in various biological processes could be associated with the diverse functions of *Ghd2*, including the regulation of flowering time and/or drought resistance.

In addition to the CCT domain-interacting 14-3-3 proteins, OsPURα and OsARID3 specifically interact with the B-box domain of Ghd2. OsPURα is a DNA/RNA-binding protein (Chang *et al.*, 2011), and it may facilitate the transcriptional regulation of Ghd2 via its DNA-binding activity. Interestingly, we also identified OsARID3 as a Ghd2-interacting protein. OsARID3 is a member of the rice AT-rich interaction domain (ARID) family, which was identified as being required for shoot apical meristem development (Xu *et al.*, 2015). *ICL* and *MS*, which were found to be up-regulated in the *Ghd2*-OE plants, were down-regulated in the *OsARID3*-OE plants, suggesting that Ghd2 and OsARID3 may work antagonistically in regulating the genes associated with senescence.

We noticed that the expression profiles of *OsPURα* and *OsARID3* were different from that of *Ghd2*. *OsPURα* showed no obvious transcript level change in the drought stress treatment, but its expression pattern was similar to *Ghd2*, with the highest level at the grain-filling stage, indicating that *OsPURα* may participate in the regulation of developmental senescence along with *Ghd2*. However, *OsARID3* was induced by drought stress treatment, and it was also expressed in rice leaves with the highest level at the grain-filling stage, indicating that *OsARID3* may be involved in both developmental and drought-induced leaf senescence mediated by *Ghd2*.

In conclusion, Ghd2 is a CO-like transcription factor which affects drought sensitivity by accelerating drought-induced leaf senescence. Ghd2 is also involved in the regulation of developmental and dark-induced leaf senescence. The accelerated leaf senescence exhibited by *Ghd2* overexpression can be partially explained by the regulation of several SAGs, including *ICL*/*Osl85*, *MS*, *OsPPDKB*, *OsAS1*, *OsSGR*, *OsNYC3*, and *OsPAO*. The interaction proteins of Ghd2, including OsARID3, OsPURα, and 14-3-3 proteins, could participate in the Ghd2-mediated regulation network in developmental and stress-induced leaf senescence.

## Supplementary data

Supplementary data are available at *JXB* online.

Table S1. Primers used in this study.

Table S2. Public microarray results of *OsK*, *OsL*, and *OsJ* in response to drought stress treatment.

Table S3. Public microarray results of *OsK* in response to drought stress treatment in three rice organs.

Table S4. GO analysis of up-regulated DEGs in the *Ghd2*-OE plants.

Figure S1. Expression level and copy number detection.

Figure S2. Phenotypes of the *Ghd2*-OE plants.

Figure S3. Phenotypes of the *Ghd2*-OE and *Ghd2*-CRISPR plants under drought stress treatment.

Figure S4. Stomata and water loss rate in the *Ghd2*-OE and WT' plants.

Figure S5. H_2_O_2_ content in the *Ghd2*-CRISPR and WT plants under drought stress treatment.

Figure S6. Diurnal expression levels of *Ghd2*, *OsPURα*, *OsARID3*, and *14-3-3* proteins at different developmental stages.

Figure S7. Ghd2 is located in the nucleus and functions as a transcriptional activator.

Supplementary Data
