# Supplementary material for: Ghd2, a CONSTANS-like gene, confers drought sensitivity through regulation of senescence in rice
Source: J Exp Bot. 2016 Sep 16;67(19):5785–98. doi: 10.1093/jxb/erw344 (PMC5066496; doi:10.1093/jxb/erw344)
Supplement: Supplementary Data [file supp_67_19_5785__index.html]

Ghd2, a CONSTANS-like gene, confers drought sensitivity through regulation of senescence in rice — Ghd2, a CONSTANS-like gene, confers drought sensitivity through regulation of senescence in rice — Supplementary Data 

# *Ghd2,* a *CONSTANS*-like gene, confers drought sensitivity through regulation of senescence in rice

## Supplementary Data

Data files

- Supplementary\_Tables\_S1\_S4\_Figures\_S1\_S7.pdf - Supplementary Data
