# Supplementary material for: Ghd2, a CONSTANS-like gene, confers drought sensitivity through regulation of senescence in rice
Source: J Exp Bot. 2016 Sep 16;67(19):5785–98. doi: 10.1093/jxb/erw344 (PMC5066496; doi:10.1093/jxb/erw344)
Supplement: Supplementary Data [file supp_erw344_Supplementary_Tables_S1_S4_Figures_S1_S7.pdf]

Supplementary Tables and Figures

Supplementary Table S1. Primers used in this study.

Name	Forward primer	Reverse primer	Purpose
Ghd2-OE	*ggggtacc*CTGGGAGATTTCTTCTGTTT	*cgggatcc*TCGAGTCCATGAGGCATA	Overexpression
Ghd2-flag	*ggggtacc*CTGGGAGATTTCTTCTGTTT	*cgggatcc*GGTGACGGCGCACGGGGC	Overexpression (3×flag)
Ghd2-sub	*cgcggatcc*CTGGGAGATTTCTTCTGTT	*cgcggatcc*CTTGACGAACCGGCCCTT	Subcellular localization
Ghd2-grna	*ggca*ATCCTCATCGTGCTTCAAGG	*aaac*CCTTGAAGCACGATGAGGAT	CRISPR
Ghd2-cas-PCR	TTTGTGCTGGGAGATTTCTT	GCCTCGTAGTTGAGCCTGA	PCR for sequencing
Ghd2-cas-seq	TCGGTGGAGGAGGAGGCG		Sequencing
CAS9-PCR	GCATGAAGAGGATCGAGGAG	GATCTCTTGCTCGGACTTGG	*CAS9* detection
HPT-PCR	TACACAGCCATCGGTCCAGA	TAGGAGGGCGTGGATATGTC	*HPT* detection
Ghd2-probe	ATGGTCGAAGCGCAA	ACAACCCCGAGTAATGG	Northern
Ghd2-BiFC	*cgcggatcc*CTGGGAGATTTCTTCTGTT	*ggggtacc*GGTGACGGCGCACGGGGC	BiFC
GF14b-BiFC	*cgggatcc*GGCATTTGTAGAGTTTTTAGAT	*cgggatcc*CTGCCCCTCGCTGGAGT	
GF14c-BiFC	*cgggatcc*TAATCCCTTAATTGGTCAAA	*cgggatcc*CTGGCCCTCGCAGGCGT	
OsARID3-BiFC	*cgggatcc*GCATCTGGATTTGAGCCTA	*cgggatcc*CATTAACTTTGACTGCTCGAA	
Ghd2-Y2H	attB1TGAGCTGCAGCTCGGAGAA	*att*B2-TCGAGTCCATGAGGCATACG	Y2H
Ghd2-Y2H-N1	*att*B1-TGAGCTGCAGCTCGGAGAA	*att*B2-*cctaCTCCTCCACCGACGCC*	
Ghd2-Y2H-N2	*att*B1-TGAGCTGCAGCTCGGAGAA	*att*B2-*ccta*TGCGGCCGCCGCGTCATC	
Ghd2-Y2H-N3	*att*B1-TGAGCTGCAGCTCGGAGAA	*att*B2-*ccta*ATCCGGGAGCTGCTGCAC	
Ghd2-Y2H-N4	*att*B1-TGAGCTGCAGCTCGGAGAA	*att*B2-*ccta*CAGCTCGTTGCTGTCGTC	
Ghd2-Y2H-N5	*att*B1-TGAGCTGCAGCTCGGAGAA	*att*B2-*ccta*GCTGTTGGAGGCAACACC	
Ghd2-Y2H-N6	*att*B1-TGAGCTGCAGCTCGGAGAA	*att*B2-*ccta*CACCGTCATCATCCCCAC	
Ghd2-Y2H-C1	*att*B1-TGGGGATGATGACGGTGA	*att*B2-TCGAGTCCATGAGGCATACG	

Ghd2-Y2H-C2	*att*B1-GGGTATCTCTCCCATCAT	*att*B2-TCGAGTCCATGAGGCATACG	
Ghd2-Y2H-C3	*att*B1-TGCAGGACTCGTTCTACA	*att*B2-TCGAGTCCATGAGGCATACG	
Ghd2-Y2H-C4	*att*B1-GCTTTGTCAACTTCGAAC	*att*B2-TCGAGTCCATGAGGCATACG	
Ghd2-Y2H-C5	*att*B1-CCTCATCGTGCTTCAAGG	*att*B2-TCGAGTCCATGAGGCATACG	
Ghd2-Y2H-C6	*att*B1-CGGCGGCGGCGGTGGGGA	*att*B2-TCGAGTCCATGAGGCATACG	
Ghd2-none	*cgggatcc*CTGGGAGATTTCTTCTGTTT	*ggggtacc*TCGAGTCCATGAGGCATA	
Ghd2-gal4BD	*cgggatcc*AGCTGCAGCTCGGAGAAG	*ggggtacc*TCGAGTCCATGAGGCATA	
ICL-190	*cccaagctt*GAAACGAGAACTACGACGAGC	*gaagatct*CACGCACTGTCAACGATGAG	
MS-190	*cccaagctt*TCTGTGAGACAGATAGGTGGA	*gaagatct*GTGTGAGTAGAAAATGGGAGT	
OsPPDKB-190	*gaagatct*CCGTGTTTCACTGTTTCCCAATGTC	*gaagatct*CGATCTCCCCTCCAACTCCAAGC	LUC assay in protoplasts
OsAS1-190	*cccaagctt*GACAAATACGCTGAATGCATA	*gaagatct*GCCGCGAGGATTACTACC	
OsSGR-190	*cccaagctt*CGCTCTCGTTCATCCTGTT	*gaagatct*GCCTAACAACCAAAACGACTC	
OsNYC3-190	*cgggatcc*AATCGTAGACCTCAAAACCC	*cgggatcc*CAGGAAGAAAAAAAGACATAGC	
OsPAO-190	*cccaagctt*GCCTGCACCAGGGTAAAG	*gaagatct*GAAGAAGAGAGTAGGAAACGGA	
Ghd2-qRT	TTGCCTCCAACAGCAGGG	GCTTCGGATGAGCGCG	
ICL-qRT	GACAGGGCCTGGAAGTGAGA	CCTCTGAATAATCCTCATCACATCCT	
MS-qRT	ACTTCTCCGAGTACTTTGCTCATACTT	TCTAGTGCAAACAAACAGGAATTGT	
OsAS1-qRT	TGTGCCGTTCCTCGACAA	TTCCACTCAGGGTCCATGCT	
OsSGR-qRT	TTCGTTTGGTTGCCATGGTA	AGCACGCAGCGTCATTTG	qPCR
OsNYC1-qRT	GTCGTCTGCGCATTCATAATTC	GGGATCATGTGCCTGGAAGA	
OsNYC3-qRT	TGTGTGCTCCAAAGGGACAA	CCCTGCCTTTGGCACCTA	
Ubi-qRT	AACCAGCTGAGGCCCAAGA	ACGATTGATTTAACCAGTCCATGA	

Supplementary Table S2. Public microarray results of *OsK*, *OsL*, and *OsJ* in response to drought stress treatment at 7-day-old seedling stage.

Name	Normal growth			Drought stress		
	Replicate 1	Replicate 2	Replicate 3	Replicate 1	Replicate 2	Replicate 3
OsK	277.1	264.6	332.5	154.2	139.7	155.4
OsL	156.8	185.6	209.3	15.2	18.5	20.9
OsJ	828.5	780	1075	373	319.9	360

Supplementary Table S3. Public microarray results of *OsK* in response to drought stress treatment in three rice organs.

Organ	Drought-1		Drought-2		Drought-3	
	LogRatio	*P*-value	LogRatio	*P*-value	LogRatio	*P*-value
Flag leaf	-0.317	2.35E-02	-0.233	1.58E-01	-0.805	2.15E-02
Shoot	-0.242	2.79E-01	-0.016	8.49E-01	-0.975	1.10E-04
Panicle	N/A	N/A	N/A	N/A	-1.221	3.58E-02

Supplementary Table S4. GO analysis of up-regulated DEGs in the *Ghd2*-OE plants during drought stress treatment.

GO Term		Count[a]	*P*-value
Biological process			
GO:0003333	amino acid transmembrane transport	36	1.20E-06
GO:0098656	anion transmembrane transport	65	3.10E-06
GO:0006787	porphyrin-containing compound catabolic process	25	3.70E-06
GO:0033015	tetrapyrrole catabolic process	25	3.70E-06
GO:0051187	cofactor catabolic process	28	6.70E-06
GO:0015849	organic acid transport	129	3.80E-05
GO:0046942	carboxylic acid transport	129	3.80E-05
GO:0015996	chlorophyll catabolic process	23	6.60E-05
GO:0046149	pigment catabolic process	24	7.90E-05
GO:0006865	amino acid transport	75	8.90E-05
Molecular function			
GO:0015171	amino acid transmembrane transporter activity	71	6.60E-05
GO:0005342	organic acid transmembrane transporter activity	105	7.50E-05
GO:0046943	carboxylic acid transmembrane transporter activity	105	7.50E-05
GO:0015293	symporter activity	126	0.00023
GO:0008514	organic anion transmembrane transporter activity	131	0.0003
GO:0015294	solute:cation symporter activity	37	0.00045
GO:0016746	transferase activity, transferring acyl groups	344	0.00056
GO:0008509	anion transmembrane transporter activity	200	0.00078
GO:0016798	hydrolase activity, acting on glycosyl bonds	363	0.00087
GO:0016747	transferase activity, transferring acyl groups other than amino-acyl groups	308	0.00089
Cellular component			
GO:0000329	fungal-type vacuole membrane	14	0.00015
GO:0000324	fungal-type vacuole	15	0.00019
GO:0009570	chloroplast stroma	331	0.00024
GO:0009532	plastid stroma	335	0.00026
GO:0000322	storage vacuole	17	0.00028
GO:0044434	chloroplast part	651	0.00151
GO:0009536	plastid	1784	0.00202
GO:0044435	plastid part	686	0.0024
GO:0009507	chloroplast	1755	0.00349
GO:0000323	lytic vacuole	88	0.00478

Note: The listed terms is top ten overrepresented GO terms according to the *P*-value.

[a] The number of differential expressed genes fall in each GO in the *Ghd2*-overexpressing lines.

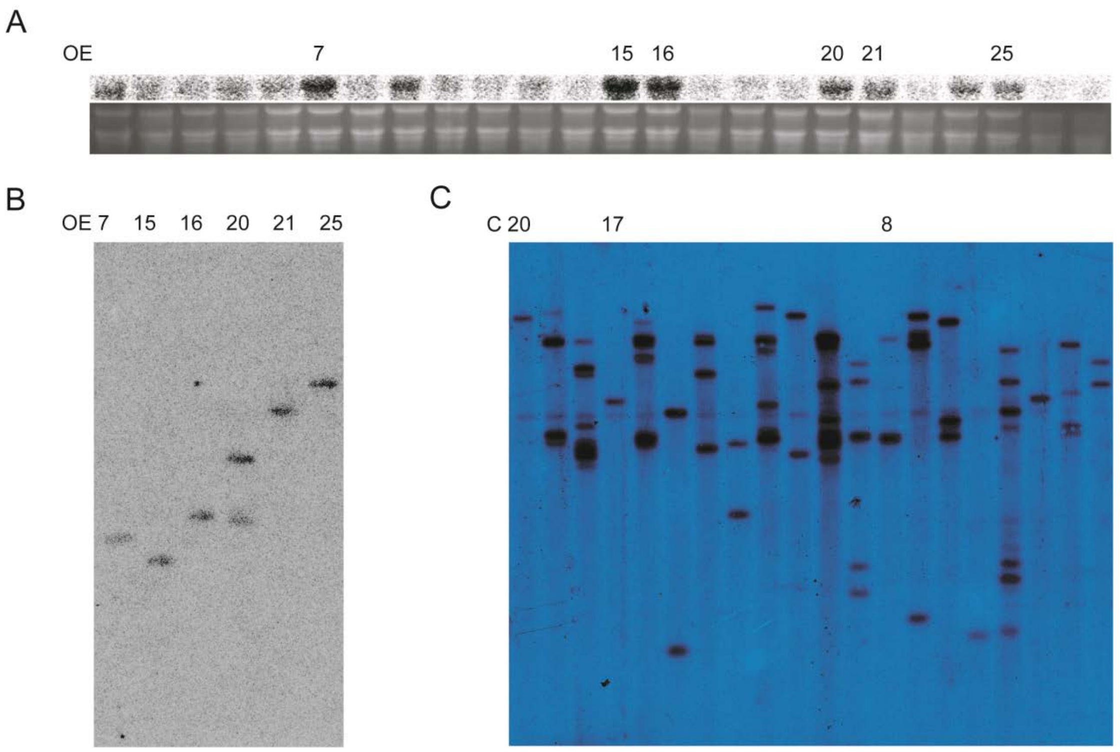

Supplementary Figure S1. Expression level and copy number detection by Northern and Southern blot respectively. Southern blot analysis used hygromycin resistance gene as a probe. (A) The *Ghd2*-OE lines were identified by Northern blot analysis. (B) Copy number of the *Ghd2*-OE lines detected by Southern blot analysis. (C) Copy number detection of the *Ghd2*-CRISPR lines by Southern blot analysis. Probes of (A) and (B) were radio-labeled, and probe for (C) was digoxigenin-labeled.

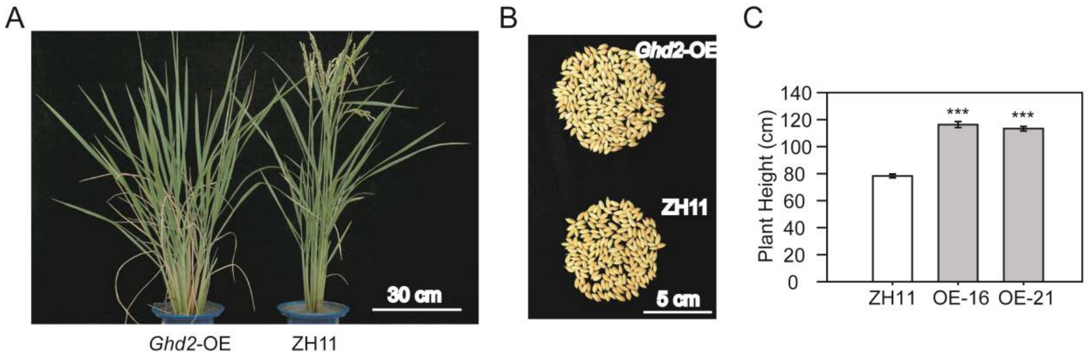

Supplementary Figure S2. Phenotypes of the *Ghd2*-OE plants with respect to grain number, plant height, and heading date. (A) Phenotypes of the *Ghd2*-OE and ZH11 plants on heading date. (B) Phenotypes of the *Ghd2*-OE and ZH11 plants on grain number per main panicle. (C) Plant height of the *Ghd2*-OE and ZH11 plants. Data represent the mean ± SE (n=9). ****P*<0.005, *t*-test.

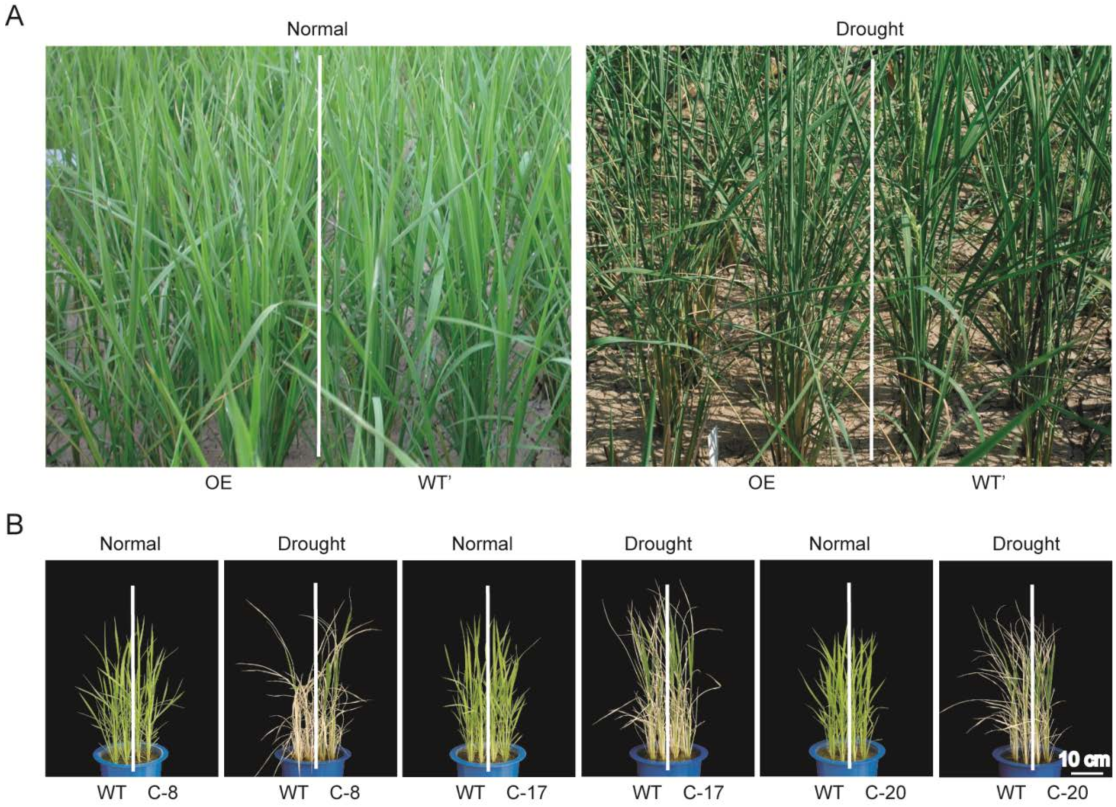

Supplementary Figure S3. Phenotypes of the *Ghd2*-OE plants at 60 DAG and the *Ghd2*-CRISPR plants at the seedling stage under drought stress treatment. (A) Phenotypes of the *Ghd2*-OE21 plants at 60 DAG under drought stress treatment in the field. (B) Phenotypes of the *Ghd2*-CRISPR plants at the seedling stage under drought stress treatment.

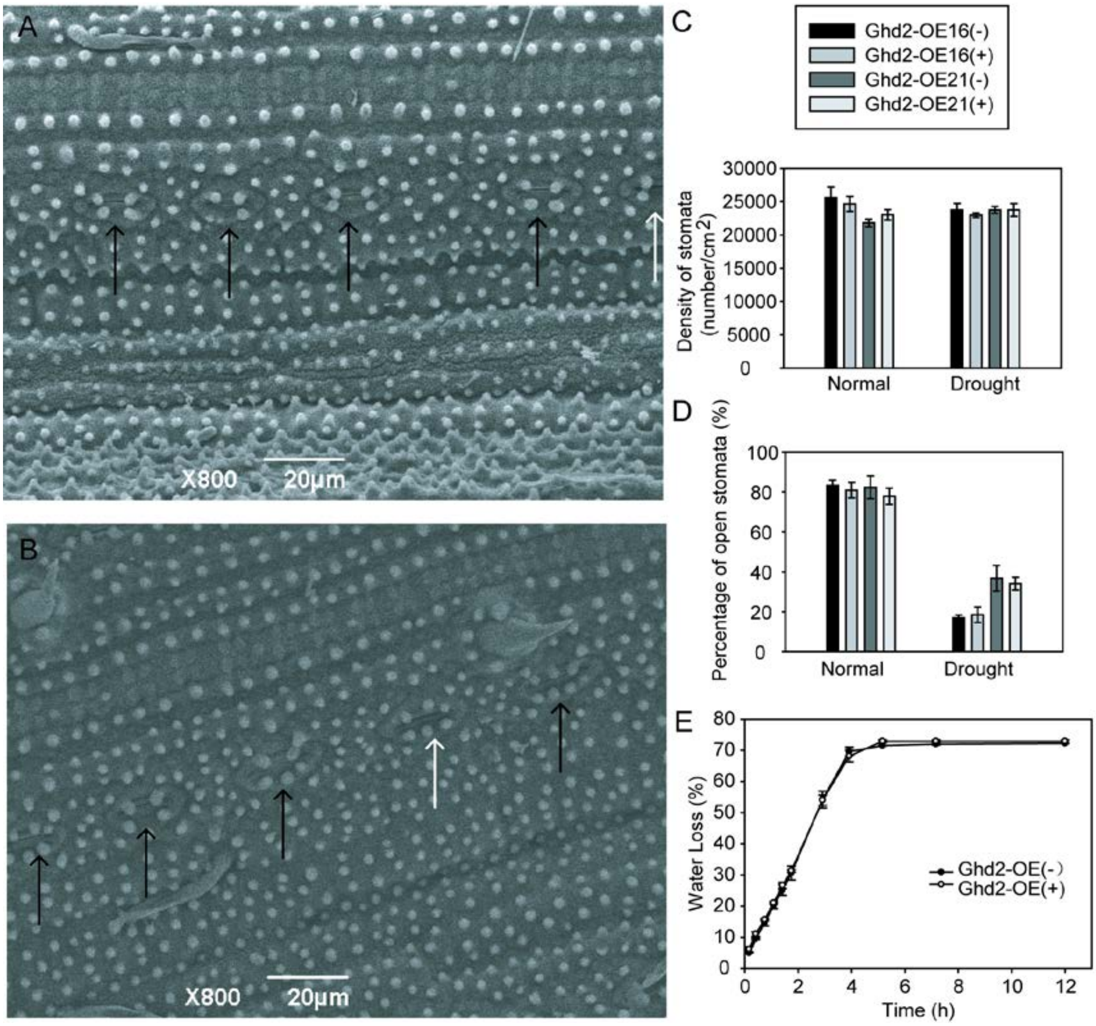

Supplementary Figure S4. Stomata and water loss rate in the *Ghd2*-OE and WT' plants. (A-B), SEM images of the *Ghd2*-OE (A) and WT' (B) plant leaves under drought stress. The open and closed stomata are indicated by white and black arrows respectively. (C-D), Stomata density (C) and percentage of open stomata (D) in the overexpression (Ghd2-OE (+)) and WT' control (Ghd2-OE (-)) plant leaves under drought stress. (E) Water loss rate in detached rice leaves of the *Ghd2*-OE and WT' control plants (n=3).

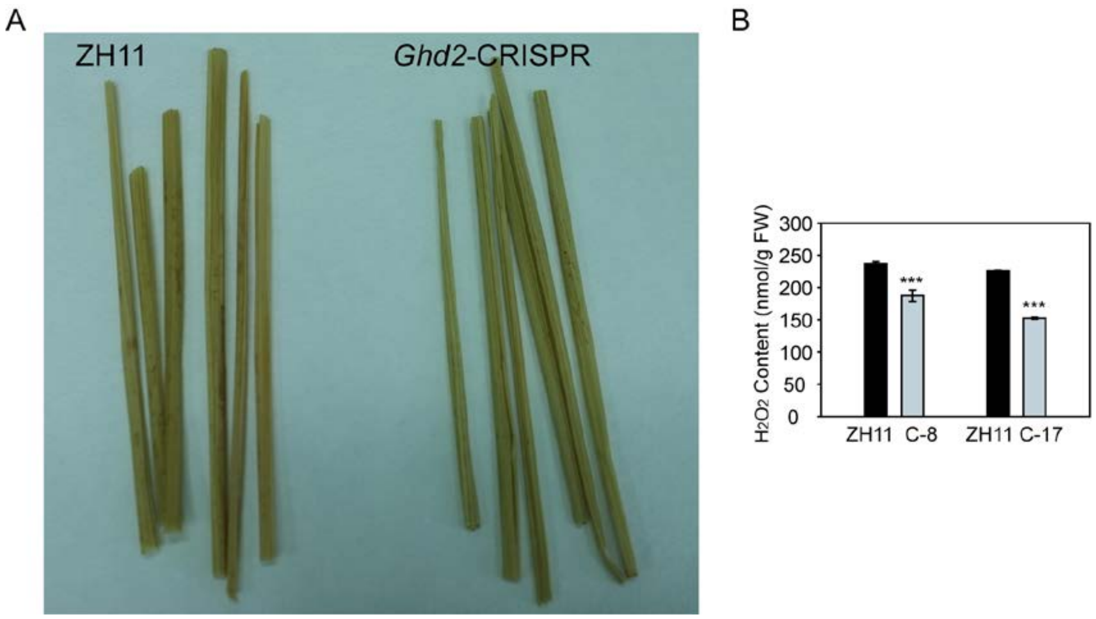

Figure S5. H_2O_2 content in the *Ghd2*-CRISPR and WT plants under the drought stress treatment. (A) DAB staining of leaves in the *Ghd2*-CRISPR and ZH11 plants during the drought stress. (B) H_2O_2 content measurement in the leaves of the *Ghd2*-CRISPR lines (C-8 and C-17) and ZH11 plants during the drought stress. ****P*<0.005, *t*-test. FW, Fresh Weight.

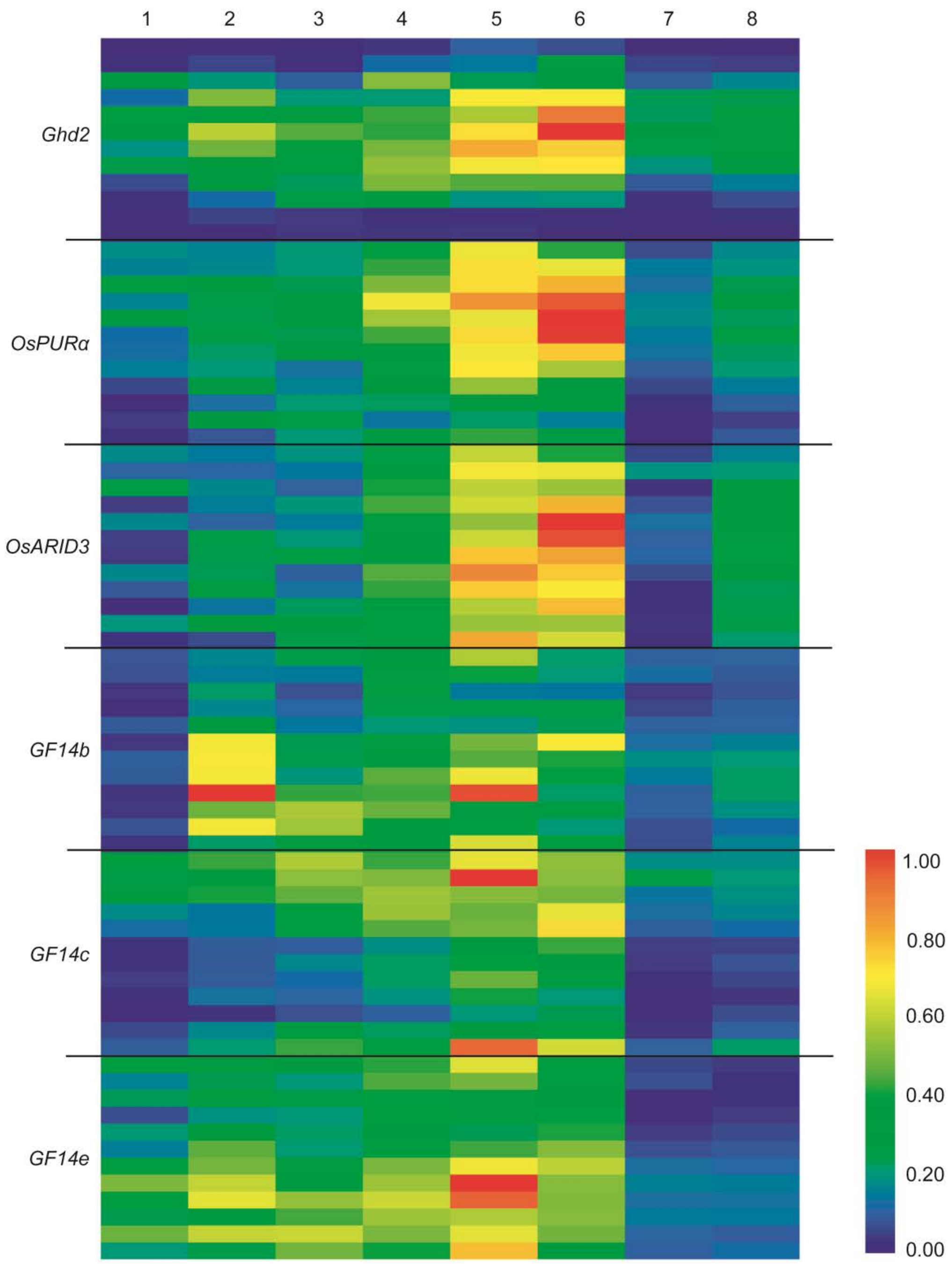

Supplementary Figure S6. Diurnal expression levels of *Ghd2*, *OsPURα*, *OsARID3*, and *14-3-3s* in rice leaves at different developmental stages. Numbers from 1 to 8 in the *x*-axis refer to "vegetative 1", "vegetative 2", "vegetative 3", "vegetative reproductive", "reproductive 1", "reproductive ripening", "ripening 1", "ripening 2", respectively (as named in the website), and the *y*-axis indicates time points of each gene from am10:00 to am 8:00. Original data was obtained from rice expression database (http://ricexpro.dna.affrc.go.jp/) and analyzed using Heatmap Illustrator.

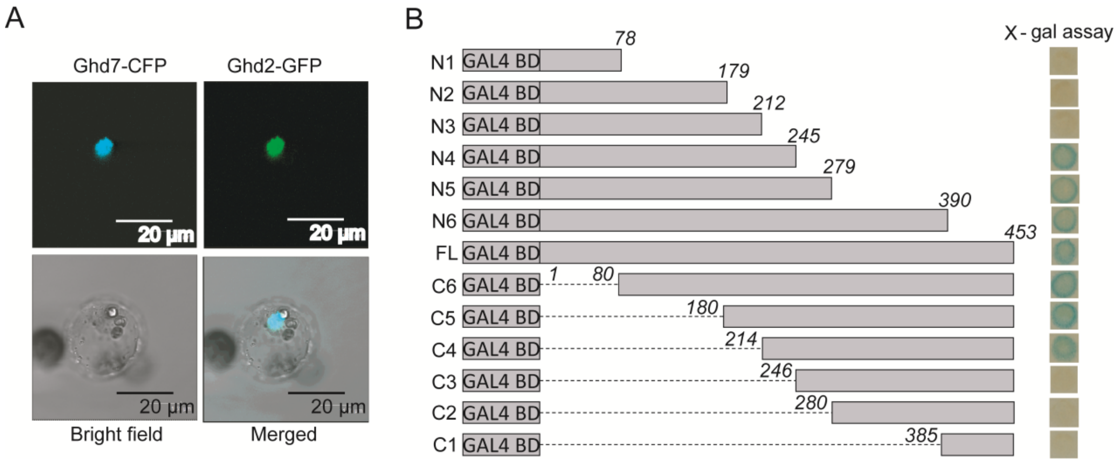

Supplementary Figure S7. Ghd2 is located in the nucleus and functions as a transcriptional activator. (A) Nuclear localization of Ghd2 in rice protoplast. Nuclear localized CCT domain containing protein Ghd7 was used as a maker to show the nucleus. (B) The activation assay of a series of deletion mutants of Ghd2 in yeast to show the transactivation activity.
